# TFE3-associated perivascular epithelioid cell tumor with complete response to mTOR inhibitor therapy: report of first case and literature review

**DOI:** 10.1186/s12957-021-02462-5

**Published:** 2022-03-01

**Authors:** Roli Purwar, Kishan Soni, Mridula Shukla, Ashish Verma, Tarun Kumar, Manoj Pandey

**Affiliations:** 1 Department of Surgical Oncology, Institute of Medical Sciences, Banaras Hindu University, Varanasi, India; 2 Department of Histopathology, Lal path labs, Varanasi, India; 3 Department of Radiology and Imaging, Institute of Medical Sciences, Banaras Hindu University, Varanasi, India

**Keywords:** Perivascular epitheliod cell tumor, Mtor Therapy, TFE3, PEComa, Uterus, Gynecological

## Abstract

**Background:**

Perivascular epitheloid cell tumor (PEComas) are characterized by expression of both muscles, most often smooth muscle actin (in ~80% of cases) and melanocytic markers (mainly HMB-45 and Melan A). TFE 3-associated PEComas are new variant which are poorly defined due to their limited reports in literature. These tumors lack response to targeted mTOR inhibitor therapy due to lack of mutation in TSC gene. Hereby, we are reporting a case of TFE3 associated pelvic PEComa showing excellent response to Everolimus.

**Case presentation:**

A 45-year-old female presented with complaint of abdominal mass and bleeding per vaginum for 4 months. She had a history of total abdominal hysterectomy 3 years back in view of abnormal uterine bleeding and exploratory laprotomy 7 months back to remove some pelvic mass. Imaging suggested of ill-defined heterogenous mass of 9.3 x 9.2 x 16 cm involving the uterus, cervix, and upper 1/3 vagina. Multiple omental and peritoneal deposits were also seen, making probable diagnosis of carcinoma endometrium. USG guided biopsy showed cores of fibrous tissue with the presence of cells in sheets with granular eosinophillic cytoplasm; IHC showed positivity for TFE-3, H Caldesmon, GATA-3, and Melan A- and HMB-45; and Ki 67 index was 35%. The basis of above diagnosis of PEComa was made and she was started on Everolimus; repeat imaging after 3 months of therapy showed complete response.

**Conclusion:**

We are reporting first case of malignant pelvic TFE 3 PEComa showing response to mTOR therapy. Identification of TFE 3 PEComa is important because they showed different biologic behavior then their conventional PEComa.

## Background

WHO defines perivascular epitheloid cell tumor (PEComas) as “mesenchymal tumors composed of histologically and immunohistochemically distinctive perivascular epithelioid cells,” including angiomyolipoma, clear cell sugar tumors of the lungs, lymphagioleiomyomatosis, hepatic falciform ligament clear cell myomelanocytic tumor, and other unusual clear cell tumors at various locations [1]. PEComa tumors are characterized by expression of both muscles, most often smooth muscle actin (in ~80% of cases) and melanocytic markers (mainly HMB-45 and Melan A), which is one of the main characteristic feature [2]. These tumors usually show female preponderance with a mean age of presentation around 45 years. Anatomically, these have a ubiquitous appearance, with most common site of origin being the kidney, lung, and liver. The uterus is the most common site for genitourinary tract PEComa [3].

PEComas are initially divided into three categories by Folpe et al. as benign (no atypical features), uncertain malignant potential (nuclear atypia or size> 5cm) and malignant by any 2 morphological and pathological criteria such as gross size (>5cm), high nuclear grade, necrosis, vascular invasion, or a mitotic rate higher than one per 50 HPF [4]. Schoolmeester later classified these lesions as malignant when these lesions meet four out of the five above mentioned criteria [5].

In a largest case series reported till now by Bennet et al. of 32 uterine PEComas, most common clinical presentation was non-specific like menstrual complaints (33%), pelvic mass/adnexal mass/uterine mass (17%), presumed fibroids (17%), metastasis from uterine primary (7%), and cervical polyp (3%). At the time of disease reporting, metastasis was found in 17% cases with most common site being the lung [6]. Commonly, pre-operative diagnosis of PEComa is rare due to the presence of nonspecific imaging features. These lesions are usually confused with leiomyomas and leiomyosarcomas [6]. PEComas are usually sporadic, but 6% of cases are associated with tuberous sclerosis with mutation in TSC1 and TSC 2 gene [7].

FTFE 3 or transcription factor binding to IGHM enhancer 3, associated PEComa, are newly defined varieties of PEComa. Around 20% of PEComas were found to be positive for TFE3 nuclear staining, among which many of them harbour TFE3 gene rearrangement. TFE-3 is a member of microphthalmia-associated transcription (MiT) family of transcription factors, which includes MITF, TFE-3, TFE B, and TFEC gene located at chromosome Xp11.2. MiTF-TFE family assist in the development of melanocytic cells. These tumors strongly express diffuse positiveness for HMB-45 and TFE-3, whereas it is weakly positive or negative for SMA and negative for melan A. TFE 3-associated PEComas can be associated with prior history of chemotherapy [8].

Other tumors which similarly expresses TFE 3 gene are melanoma, clear cell sarcoma, alveolar soft part sarcoma, and translocation-associated renal cell carcinoma. These tumors are considered as microphthalmia-associated transcription factor family of tumors [9].

In this article, we report a case of TFE 3 positive uterine PEComa, with a mixed cell pattern of both epitheliod and spindle cells, which strongly express HMB-45, Melan A, SMA, and surprisingly responds to everolimus (mTOR inhibitor).

## Case report

A 45-year-old female presented to surgical oncology outpatient with a chief complaint of abdominal mass and per vaginal bleed for 4 months. As stated by the patient, she had undergone an abdominal surgical exploration before 7 months to remove pelvic mass. The patient also gave history of total abdominal hysterectomy before 3 years in view of abnormal uterine bleeding. However, the patient did not have any documentation regarding the abovementioned surgeries. The patient did not have any significant personal and family history. Clinical examination general condition was fair; vitals were stable; pallor was present; on per abdominal examination, a large, soft abdominopelvic mass of 15 x 15 cm in size was found, which was fixed, non-tender, not moving with respiration and was more deviated towards the left side. On per speculum examination, the vault was found replaced by a big smooth growth occupying upper 2/3rd of the vagina, but no vaginal invasion was present. On per vaginum, a smooth lobular abdominopelvic mass of around 15 x 15 cm was felt arising from vault. Routine biochemical investigations were within normal limit, except for hemoglobin level, which was 9gm%. An MRI was done which showed ill-defined heterogeneously enhancing mass lesion noted involving the uterus and cervix, measuring 9.3 x 9.2 x 16 cm (AP X TR X CC). The lesion was invading adjacent myometrium, reaching up to serosa in the fundal region. It was also infiltrating the bilateral adnexa, but bilateral ovaries were not separately visualized. Inferiorly involvement extended till the upper 1/3 of the vagina. Anteriorly, the lesion was closely abutting the urinary bladder with suspicious loss of fat planes at places. Enlarged and necrotic common iliac lymph nodes were noted, largest measuring 1.1 cm. Multiple omental and peritoneal deposits were noted, largest measuring 4.2 x 2.9 cm. There was no ascites and blood inside the peritoneal cavity. MRI suggested a probable diagnosis of endometrial carcinoma with extensions (Fig. 1.1 A). An USG-guided biopsy was taken from the pelvic mass, which on histopathological examination, showed cores of fibrous tissue along with the presence of cells in sheets, micro-papillae, perivascularly arranged, and were polygonal with well-defined cytoplasmic borders, granular eosinophilic cytoplasm, central to eccentric round nuclei (Fig. 2a–c). Few cells showed nuclear inclusion, occasional psammomatous calcification which suggest of neoplastic etiology. An immunohistochemistry (IHC) panel was requested which revealed TFE-3 score 3+, H Caldesmon score 1+, GATA-3 score 1+, Melan A-score 4+, and HMB-45, score 4 +. Ki 67-positivity was seen in 30–35% of tumor cells (Fig. 3a–d). On the basis of histopathological and IHC analysis, the diagnosis of perivascular tumor with melanocytic differentiation was made and the possibility of TFE 3 mutation associated with PEComa was favoured. In view of advanced nature of disease, and due to rarity of cases with no standard guidelines for the management of uterine pecoma, decision for chemotherapy was taken and the patient was started on mTOR inhibitor, everolimus 10 mg OD. She was kept on monthly follow-up, in every visit, she was symptomatically improved. After 3 months of therapy, she was again examined, and there was no mass/lump palpable, on per abdomen and per vaginum examination. Repeat MRI pelvis was done which showed nearly resolution of pre-existing mass with decreased signal intensity showing nonviable remnants (Fig. 1.1 B). As per RECIST (Response Evaluation Criteria in Solid Tumors) criteria, tumor shows partial response with mTOR inhibitors within 3 months of therapy. She was again followed after 6 months of therapy, there was again no mass/lump palpable on examination, MRI was repeated again showing no evidence of any solid mass lesion or altered enhancement noted, and urinary bladder (straight long arrow) and rectum (straight short arrow) can now be clearly seen without compression in the pelvis (Fig. 1.2), suggesting a complete response as per RECIST criteria. Multiple ROI (region of interest) values were acquired at anatomically distinct areas of the tumor before and after CT on MRI. The correlation of ROI over serial scans was done by an approximate visual assessment. It was noted that there was elevation of ADC (apparent diffusion coefficient) in pre- and post-chemotherapies MRI (819.2mm^2^/s-951 mm^2^/s), indicating towards good response (viz. facilitation due to apoptosis and increased vasogenic edema; however, the last scan showed reduction in mean ADC values (789mm^2^/s), most probably due to overall tumor volume on ROI becomes negligible and probably due to post CT fibrosis. Till now date patient is following with us, and she is continuing her everolimus, with no recurrence of lesion after 1 year of therapy.

**Fig. 1:**
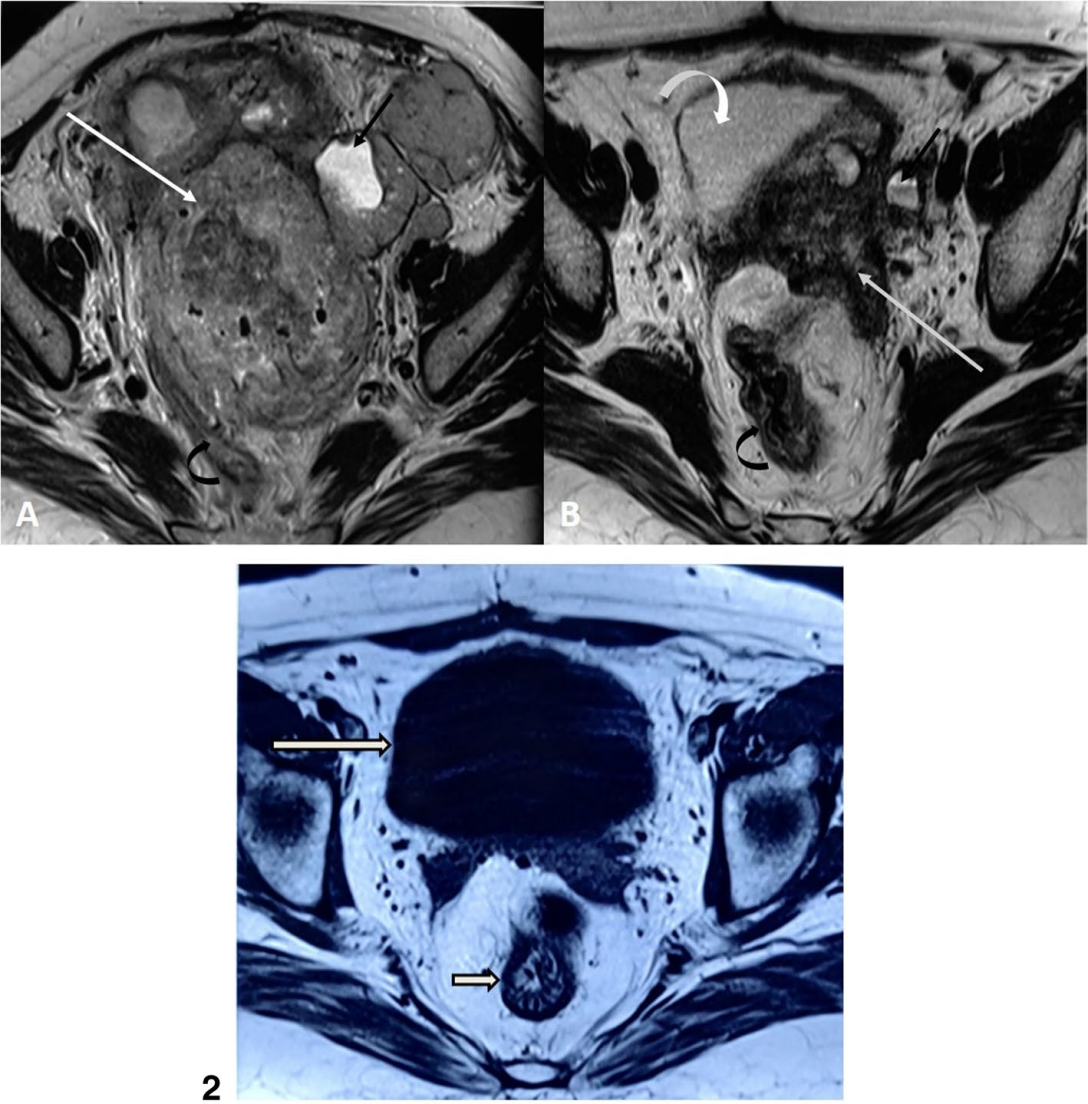
(1) Pre-chemotherapy (**A**) and post-chemotherapy (**B**) axial MRI sections of the pelvis at the level of the third sacral vertebrae. Sequences have been taken with high TE and TR values (viz. T2 weighting). The pretherapy scan (**A**) shows the bulky multilobulated mass (straight white arrow) with multiple satellite lesions (straight black arrow). On post therapy scan after 3 months (**B**), the main lesion shows near complete regression (straight white arrow) while the satellite lesions which were showing cystic degeneration (straight black arrow) have converted to much smaller hemorrhagic cysts (note the fluid-fluid level within the cyst). Also note that the urinary bladder (curved white arrow) and the rectum (curved black arrow) which were grossly compressed by the mass in the pre-therapy scan could be well visualized in the post therapy scan, confirming the regression of the tumor. Also note the reduction of signal intensity in after treatment signifying that the remaining tissue may just be the non-viable fibrotic tumoral remnant. (2) On post therapy scan after 6 months, there was no evidence of any solid mass lesion or altered enhancement noted, urinary bladder( straight long arrow) and rectum(straight short arrow) can now be clearly seen without compression.

**Fig. 2:**
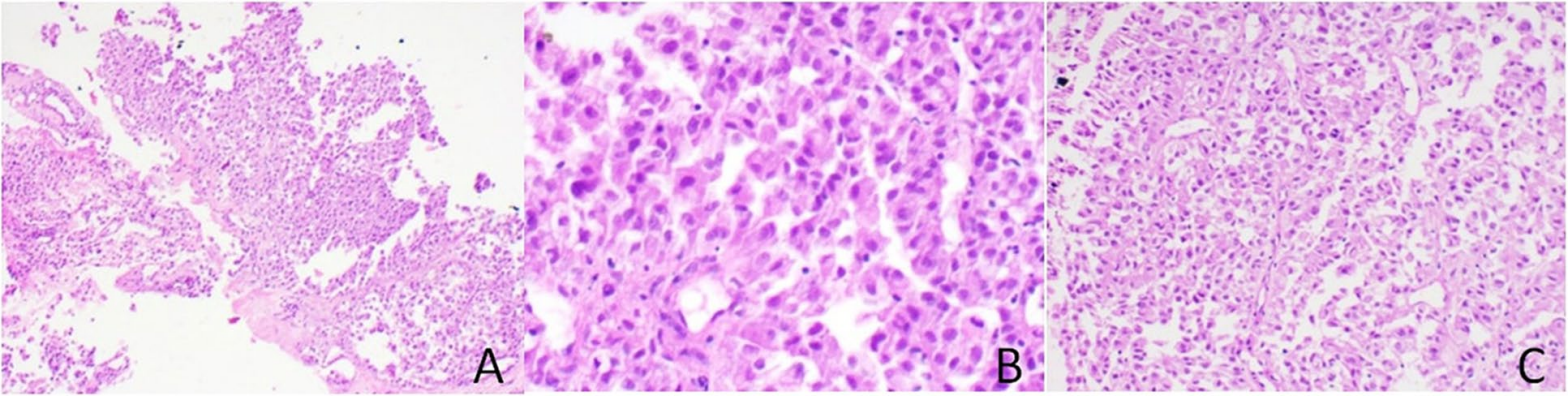
On histopathological examination. **A** The cores of the fibrous tissue along with the presence of cells in sheets; **B** 3 the polygonal cells with well-defined cytoplasmic borders, granular eosinophilic cytoplasm, and central to eccentric round nuclei; and 3 **C** the presence of cells in sheets, micro-papillae, and perivascularly arrangement

**Fig. 3:**
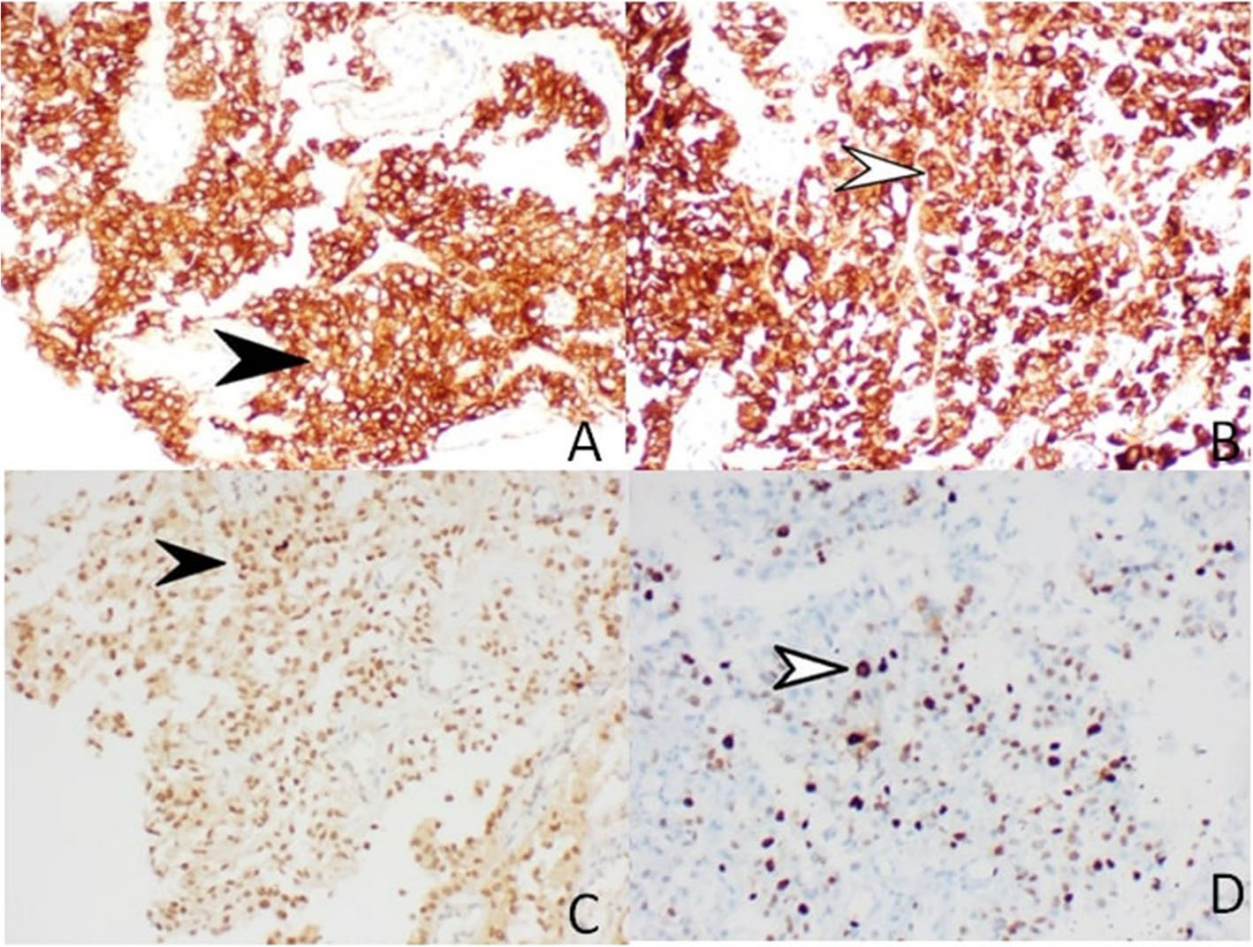
**A** Strong expression of HMB-45, Score 4+ (40x); **B** strong expression of MELAN A Score 4 + (40x); **C** Strong expression of TFE-3, Score 3 + (40x); and **D** -Ki-67 positivity seen in 30–35% of tumour cells

## Discussion and conclusions

Uterine PEComas is a rare entity, on which around 150 cases have been reported in English literature till now. There are two distinct patterns of identified in PEComas—first with epithelioid pattern (100% cases)—these are polygonal cells with clear to granular cytoplasm and positive for melanocytic markers HMB45, Melan-A, and MITF. The second being the spindle cell pattern (37% of cases), which consists of cells with less cytoplasm, arranged in fascicles like the smooth muscle and are positive for SMA, desmin, and caldesmon. HMB-45 is the most sensitive marker being positive in 100% cases. The diagnosis should always be differentiated from smooth muscle tumors of the uterus and especially tumors which showed similar IHC like epithelioid smooth muscle tumor of the uterus, high-grade endometrial stromal sarcoma (HGESS), GIST, and melanoma involving the uterus [10]. CD10 is diffuse and shows strong immunoreactivity in endometrial stromal tumors, and GIST shows strong CD34 staining, as well as c-Kit positivity and negative for melanocytic marker. Metastatic melanoma and/or clear cell sarcoma shows strong S-100 protein immunoreactivity of the former and their muscle marker negativity.

PEComas with TFE3 gene rearrangement have predominant epithelioid morphology with clear cells and have strongly positive staining for melanocytic markers like HMB-45 and Cathepsin K and weak or negative expression of myoid markers. Their rare variant is TFE 3 gene mixed cell PEComa with both epitheliod and spindle cell pattern. Morphologically, these showed clear to granular cytoplasm with eosinophilic cells and showed marked immune expression for HMB-45, TFE-3, Melan A, and also positive for myoid markers [5].

These lesions either represent collision between PEComa and smooth muscle tumor or PEComa with smooth muscle differentiation, which can be answered only by molecular analysis (Bennet 2018). This rearrangement can be explained by TFE3 fusing with other genes or undergoing breakage at different points along the gene [5].

In general, PEComa shows favorable prognosis [4], but TFE3-associated PEComas show aggressive behavior (52%of cases) and poor prognosis during follow-up [9, 11, 12]. Folpe et al. showed that TFE3 fusion PEComa has an invasive behavior, local recurrence, and metastasis rates of 8.7% and 20.3%, respectively [4]. Careful review of English literature revealed only 10 cases of uterine and cervix pecoma with TFE3 rearrangement. Table 1 shows its clinical profile, treatment, and follow-up status. Table 2 shows the immunohistochemical profile of uterine PEComas.

**Table 1:** Features of uterine TFE3 translocation associated PEComa

	Year	Age (years)	Site	Clinical features	Past h/o	Size (cm)	Pathology	Treatment	Follow-up	Outcome
1	Cho 2008 [13]	9	Uterus, lower uterine segment	Vaginal spotting, metastases to pelvic lymph nodes at presentation	None	5	Alveolar, epitheliod	TAH+pelvic LN dissection	ALL occured at 25 months	Died at 33 months because of ALL, no e/o recurrence of pecoma at time of death
2	Liu 2014 [14]	34	Cervix	AUB	None	9	Sheets/ alveolus/nests	Resection of cervical mass	5 months	Alive
3	Schoolmester 2015 [5]	53	Uterine corpus	AUB	None	17	Sheet like nested	Supracervical hysterectomy, RSO	**2 months:** cervix and metastases to omentum treated by radical trachelectomy, upper vaginectomy, omentectomy and adjuvant chemotherapy **11 months:** small and large intestine and intraabomdinal cavity treated by debulking and adjuvant chemotherapy	Alive; intraabdominal recurrence led to diagnosis revision from high grade LMS to pecoma; recently started sirolimus regimen
4	Schoolmester 2015 [5]	49	Uterine corpus	Uterine mass	Hodgkin lymphoma treated with ABVD chemotherapy (6 years prior)	33	Nested	TAH-BSO	25 months Recurrence -none	Alive, ned
5	Schoolmester 2015 [5]	47	Pelvis, site not identified	Pelvic pain	Morcellated supracervical hysterectomy with cellular leiomyomata (1 year prior)	8	Nested	Local excision of pelvic mass, radical trachelectomy, bso, pelvic and paraaortic lymphadenopathy, omentectomy, staging biopsies	57 months Recurrence-urinary bladder treated by excision	Alive, ned
6	Schoolmester 2015 [5]	46	Uterine corpus	Unknown	None	1	Nested	Hysterectomy	1 month Recurrence- none	Alive. Ned
7	Choi 2016 [15]	67	Uterus	AUB	Ns	6	Spindle cells	TAH+ BSO	Ns Multiple metastasis in lung and liver	Ns
8	Bennet 2018 [6]	Ns	Uterus	Ns	Ns	Ns	Nested	Ns	19 months	Alive
9	Gianella 2020 [11]	45	Uterus	Cyclic abdominopelvic pain and chronic constipation	K/c/o breast cancer, treated with quadrantectomy, axillary dissection, and radiotherapy, Followed by tamoxifen therapy for five years	4	Nested architecture with thin-walled vascular spaces and was Composed of large cells with a clear to granular eosinophilic cytoplasm, round to ovoid nucleus, and Prominent nucleoli	TLH with a bilateral salpingectomy.	2 years, no recurrence	Alive
10	Hu 2020 [16]	53	Uterine endom etrial polyp	Irregular menstruation	K/c/o ca breast, h/o MRM f/b tamoxifen x 4 years	2	Epitheliod cells with nested architecture	TLH	5months	Alive

Note: *TAH* total abdominal hysterectomy, *TLH* total laproscopic hysterectomy, *RSO* right salpingoopherectomy, *AUB* abnormal uterine bleeding, *ALL* acute lymphocytic leukemia, *LMS* liomyosarcoma, *NS* not specified, *MRM* modified radical mastectomy, *BSO* bilateral salphingoophorectomy, *NED* no evidence of disease

**Table 2:** Immunohistochemical and molecular profile of uterine PEComas

Case no	HMB 45	MELAN A	CATHEPSIN K	TFE 3	SMA	DESMIN	Caldesmon	Ki 67	FISH
1	Positive	0	Positive	Positive	0	0	ns	-	Not done
2	3+	3+	-	3+	0	-	-	2+	+
3	4+	2+	4+	4+	0	0	0	ns	+
4	4+	0	4+	4+	0	0	0	ns	+
5	4+	1+	4+	4+	0	1+	0	ns	+
6	4+	0	4+	4+	-	0	0	ns	+
7	3+	+	-	3+	+	+	-	5%	Not done
8	4+	-	4+	4+	4+	-	-	ns	PSF-TFE3
9	Positive	Ns	Positive	Positive	Focal positive	-	-	Ns	Not done
10	Positive	Positive	Positive	Positive	-	-	-	5%	+
Present case	4+	4+	-	3+	-	-	1+	30%	Not done

Note: *ns* not specified

In all the above cases reported in literature, the pelvic tumors were managed by primary surgical resection. In maximum of the abovementioned cases, diagnosis was confirmed postoperatively. Most of the cases does not contain any information regarding follow-up and further management.

No effective therapy with TFE 3 rearranged PEComa in advanced extrarenal cases have been mentioned in the literature. Chemotherapy (CT) and radiotherapy (RT) have also been reported in literature with advanced PEComa cases. Since there is paucity of cases, poor results reported with variety of treatment modalities and no randomized trial conducted, no uniform consensus has been achieved in this regard.

Both neoadjuvant and adjuvant CT (dacarbazine, ifosfamide, doxorubicin, vincristine) have been reported, but heterogenous results were achieved regarding disease progression and survival-free interval. Regarding targeted therapy, the use of mTOR inhibitors in conventional metastatic PEComas with TSC1 and TSC2 mutation has been reported in very few cases at other extrarenal sites with promising results; however, further prospective studies are needed [17]. TFE 3 rearranged PEComas do not involve TSC2 gene; thus, biologically, these tumors behave distinctly with conventional PEComa and do not respond to mTOR inhibitors [5].

Xu et al. have reported a case of gastrointestinal PEComa with TFE3 rearrangement which did not responded to Everolimus, hence they switched to anti-VEGFR2 and Apatinib, for which the tumor remained stable and the progression free survival lasted for about 7 months [18]. Another case of ovarian TFE3 reactive PEComa was reported, which did not responded to sirolimus and develop liver recurrence [19].

Although our case showed strong nuclear positivity for TFE3, due to non-affordability, the patient refused for FISH analysis to look for genetic rearrangements. In spite of TFE3 reactivity, our patient responded very well with Everolimus, in contrast to other cases reported in the literature.

Prompt identification of TFE-3 PEComa is recommended before starting their management, since these tumors show different biological behavior as compared to the conventional counterpart, which can lead to important insight into their management and the targeted therapies. Since very few cases have been reported in literature, further studies are needed to clearly define their clinical characteristics, prognosis, and management. We have reported the first case of TFE 3 reactive PEComa which showed an appreciable response to Everolimus.

## Data Availability

Not applicable as all information and data is presented in the manuscript.

## Abbreviations

PEComas: Perivascular epitheloid cell tumor
CT: Chemotherapy
RT: Radiotherapy
ROI: Region of interest
ADC: Apparent diffusion coefficient
IHC: Immunohistochemistry
RECIST: Response Evaluation Criteria in Solid Tumors
